# Exogenous Abscisic Acid Promotes Anthocyanin Biosynthesis and Increased Expression of Flavonoid Synthesis Genes in *Vitis vinifera* × *Vitis labrusca* Table Grapes in a Subtropical Region

**DOI:** 10.3389/fpls.2018.00323

**Published:** 2018-03-26

**Authors:** Renata Koyama, Sergio R. Roberto, Reginaldo T. de Souza, Wellington F. S. Borges, Mauri Anderson, Andrew L. Waterhouse, Dario Cantu, Matthew W. Fidelibus, Barbara Blanco-Ulate

**Affiliations:** ^1^Department of Agronomy, Londrina State University, Londrina, Brazil; ^2^Embrapa Grape and Wine, Bento Gonçalves, Brazil; ^3^Department of Viticulture and Enology, University of California, Davis, Davis, CA, United States; ^4^Department of Plant Sciences, University of California, Davis, Davis, CA, United States

**Keywords:** seedless table grape hybrid, berry quality, anthocyanin biosynthesis, MYB transcription factors, plant growth regulators, *S*-ABA

## Abstract

Hybrid (*Vitis vinifera* ×*Vitis labrusca*) table grape cultivars grown in the subtropics often fail to accumulate sufficient anthocyanins to achieve good uniform berry color. Growers of *V. vinifera* table grapes in temperate regions generally use ethephon and, more recently, (*S*)-*cis*-abscisic acid (*S*-ABA) to overcome this problem. The objective of this study was to determine if *S*-ABA applications at different timings and concentrations have an effect on anthocyanin regulatory and biosynthetic genes, pigment accumulation, and berry color of the Selection 21 cultivar, a new *V. vinifera* ×*V. labrusca* hybrid seedless grape that presents lack of red color when grown in subtropical areas. Applications of *S*-ABA 400 mg/L resulted in a higher accumulation of total anthocyanins and of the individual anthocyaninsanthocyanins: delphinidin-3-glucoside, cyanidin-3-glucoside, peonidin-3-glucoside, and malvidin-3-glucoside in the berry skin and improved the color attributes of the berries. Treatment with two applications at 7 days after véraison (DAV) and 21 DAV of *S*-ABA 400 mg/L resulted in a higher accumulation of total anthocyanins in the skin of berries and increased the gene expression of *CHI*, *F3H*, *DFR*, and *UFGT* and of the *VvMYBA1* and *VvMYBA2* transcription factors in the seedless grape cultivar.

## Introduction

Table grapes (*Vitis* spp.) have become an important fresh commodity in Brazil for both internal market and exportation. Over the period of 2000–2016, Brazil presented an increase of ∼150% in table grape production, reaching around 970,000 MT in 2016 (Food and Agriculture Organization [Fao], 2018). The northern region of Paraná state is one of the main areas of table grape production. The mild winter and subtropical conditions of this region permit two crops of grapes per year, which allow Brazilian growers to time their production to coincide with market windows of other countries and compete for more advantageous prices. However, in these subtropical regions, berry ripening and harvest often occur during the rainy season, which is not ideal for *Vitis vinifera* cultivars because excess rain and moisture compromise the overall quality of the berries (Biasoto et al., 2014). Therefore, Brazilian table grape production is starting to incorporate American (mostly *Vitis labrusca*) and/or hybrid (*V. vinifera* ×*V. labrusca*) grape cultivars that are better adapted to warm and rainy climates. Another disadvantage of growing table grapes in subtropical areas is that high temperatures during ripening can inhibit anthocyanin biosynthesis in the berries from *V. labrusca* and hybrid cultivars (Rybka et al., 2015). This results in inadequate fruit color, and thereby a decrease in market acceptance and the potential economic value of the commodity (Roberto et al., 2012). The seedless table grape Selection 21, a new hybrid of *V. vinifera* ×*V. labrusca* recently developed by the Grape Genetic Breeding Program of Embrapa Grape and Wine, Brazil, obtained from the cross of [Arkansas 1976 × (“Niagara White” × “Venus”)] × “BRS Linda,” is a clear example of a cultivar that lacks red color development when grown in subtropical regions.

The plant growth regulator ethephon, an ethylene-releasing agent, has long been known to improve berry color when applied at véraison (the onset of grape ripening) (Jensen et al., 1975; Roberto et al., 2013). More recently, the application of (*S*)-*cis-*abscisic acid (*S*-ABA) has also been shown to stimulate anthocyanin accumulation and thereby improve berry color (Peppi et al., 2006; Roberto et al., 2012). *S*-ABA appears to be more effective than ethephon in enhancing grape color (Peppi et al., 2006; Roberto et al., 2012) and it has other potential benefits compared to ethephon, including a shorter postharvest interval, and an exemption from tolerance in most countries. The introduction of *S*-ABA as an active ingredient in a commercial plant growth regulator (ProTone^®^) prompted many studies on *V. vinifera* cultivars under temperate climate conditions. Such studies have shown that the efficacy of *S*-ABA varies with the cultivar (Sandhu et al., 2011), the *S*-ABA concentration (Peppi et al., 2006), the time of application (Ferrara et al., 2015) and the environmental conditions (Reynolds et al., 2016).

Abscisic acid is an important regulator of ripening and anthocyanin biosynthesis in grape berries (Kuhn et al., 2013; Yang and Feng, 2015). Studies have shown that exogenous application of *S*-ABA can significantly increase the activity of a wide range of genes involved in anthocyanin biosynthesis (He et al., 2010). Most of these studies tested the effects of a single application of *S*-ABA before or during véraison. However, studies of the effects of *S*-ABA several applications at different concentrations and timings following véraison are still needed to optimize the use of this plant growth regulator in table grape cultivation (Peppi et al., 2008; Koyama et al., 2010).

In grapes, the anthocyanin biosynthesis pathway involves multiple steps that are controlled by MYB transcription factors, such as VvMYBA1 and VvMYBA2 (Rinaldo et al., 2015). In red varieties, the *VvMYBA1* gene is only expressed after véraison. Both VvMYBA1 and VvMYBA2 regulate anthocyanin biosynthesis during ripening by strictly controlling the expression of the canonical UDP-glucose:flavonoid 3-*O*-glucosyltransferase gene (*UFGT*; He et al., 2010). Determining how long grape berries are competent to induce the expression of anthocyanin biosynthetic genes may help determine the optimal time, number, and frequency of *S*-ABA applications. Currently, little is known about the potential benefits of multiple applications, which may be desirable if a single application results in an insufficient response.

The aim of the present study was to determine the effects of *S*-ABA applications at different concentrations and times on the quality and biochemical properties of berries from the seedless grape Selection 21 hybrid during three growing seasons in the region of Paraná, Brazil. We evaluated a variety of parameters including: (i) grape color development, (ii) berry phenolic profiles, and (iii) gene expression of transcriptional regulators and biosynthetic enzymes of the anthocyanin pathway after treatments with *S*-ABA. The results of this report indicate that two *S*-ABA applications during and after véraison extend the competency of grape berries to respond to ABA and induce the accumulation of anthocyanins, resulting in higher grape berry coloration.

## Materials and Methods

### Plant Material and Grapevine Growth Conditions

The study was conducted during three consecutive seasons (2013, 2014, and 2015) in a commercial vineyard located in Marialva, state of Paraná (PR), Brazil (latitude 23°29′52.8″S, longitude 51°47′58″0 W, 570 masl), using 4-year-old vines of hybrid seedless grape Selection 21 (*V. vinifera* × *V. labrusca*) grafted onto IAC 766 Campinas rootstock. According to the Köppen classification, the climate of the region is Cfa (subtropical), with an average temperature below 18°C in the coldest month (mesothermic) and above 22°C in the hottest month and an average annual rainfall of 1,596 mm. The region’s soil is classified as dystroferric red latosol (Bhering and Santos, 2008).

The vines were trained using a bilateral overhead trellis system, where vines were spaced at 2.5 m × 2.5 m (1,600 vines per hectare), and each vine had 6.25 m^2^ total canopy area. Cane pruning was performed during the 2013, 2014, and 2015 seasons and was followed by application of 3% hydrogenated cyanamide to the two apical buds to induce and standardize sprouting. The number of canes per vine was evenly adjusted to 40 (20 per arm) and the number of shoots per vine was also established to 40 (1 bearing shoot per cane). Considering that a grape bunch of the Selection 21 weighs on average 460 g, the load per vine is 18.40 kg, which represents an estimated yield of 29.44 tons/ha.

Furthermore, to avoid drifting, a non-treated vine was left as side border between two treated vines, which almost duplicated the experimental area. In each plot, all grape bunches were treated (*n* = 40 per vine or plot), and the bunch samples (*n* = 10, five per side) were collected from random positions at each side of the canopy to account for intracanopy variability. Control plants were not subjected to any treatment, instead, they were sprayed with water at the same time and following the same procedures as the *S*-ABA treatments.

### *S*-ABA Applications

The effects of applying *S*-ABA isomer at different concentrations and times were evaluated in terms of berry quality traits. ProTone^®^ (Valent BioSciences, Libertyville, IL, United States), the commercial growth regulator used in this study, has an active ingredient concentration of 100 g/L *S*-ABA.

**FIGURE 1:**
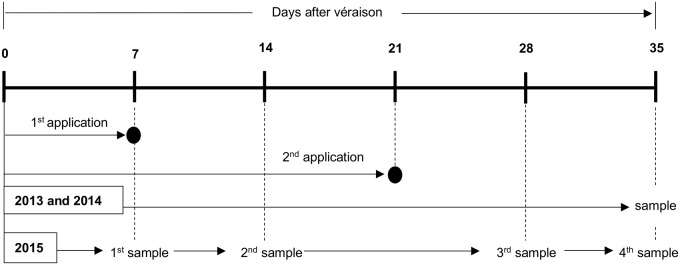
Sample collection schedule for grape clusters treated with *S*-ABA during the 2013, 2014, and 2015 seasons.

As shown in **Figure 1**, the initial treatments tested in the 2013 and 2014 seasons corresponded to: (i) control or water spray, (ii) 200 mg/L *S*-ABA application at 7 days after véraison (DAV), (iii) 400 mg/L *S*-ABA application at 7 DAV, (iv) 200 mg/L *S*-ABA application at 7 DAV plus an additional application at 21 DAV, and (v) 400 mg/L *S*-ABA at 7 DAV plus an additional application at 21 DAV. In the 2015 season, only the control and treatments of 400 mg/L *S*-ABA with one or two applications were performed and analyzed. Berry samples from the 2015 season were collected from each treatment at four different times: 7 DAV (collected 1 h before treatment application), 14 DAV, 28 DAV, and 35 DAV for further targeted analyses (**Figure 1**). For all seasons, a randomized complete block experimental design was used, with five treatments and three to four replicates, and with each plot consisting of one vine (see the previous section for details on trellis system).

Véraison was determined by measuring soluble solid content (SSC) and firmness (using a Texture Analyzer, details below) of grape berries randomly sampled in the experimental vineyard. At véraison, the mean grape SSC concentration was 9°Bx, and 20% of the berries in more than 50% of the grape clusters presented softening (Cantín et al., 2007). The berries presented a mean of 11°Bx at 7 DAV, the time of the first *S*-ABA application, and a mean of 13°Bx at 21 DAV, the time of the second *S*-ABA application.

Ten grape clusters representative of each plot were marked prior to treatment application. For treatment applications, grape clusters were sprayed in the morning using a knapsack sprayer at a pressure of 568.93 psi (39.22 bar) with JA1 hollow cone nozzle tips at a volume of 800 L/ha to provide complete and uniform coverage. In addition, 0.3 mL/L of Break-Thru^®^ (Evonik Industries, Germany) a non-ionic surfactant was added to all treatments. During the trials, the standard regional cultivation practices with regard to nutrition, weed control, and pest and disease management were used.

### Berry Physiological Measurements

Clusters of each plot were manually harvested when SSC stabilized (∼15°Bx). The clusters were cleaned, and damaged berries were discarded. Color coverage (% red color) of the bunches was determined using 10 grape clusters per plot by visually rating the clusters on a scale of 1–5 using the following scale: (1) 0–20%, (2) 21–40%, (3) 41–60%, (4) 61–80%, and (5) 81–100% coverage (Roberto et al., 2012). The same grape clusters used for evaluating color coverage measurements were used for berry sampling. For physicochemical analyses, two berries were collected from the upper, middle, and lower portion of each grape bunch, yielding a total of 70 berries per plot. Total anthocyanins and color index of red grapes (CIRG) were determined in berry samples from all seasons. The following variables were analyzed only for the 2013 and 2014 seasons: color coverage, total polyphenols, and berry firmness. All physiological analyzes were performed in the Laboratory of Fruit Analysis of the Agricultural Research Center, Londrina State University, Brazil.

The total anthocyanin concentration of the berries was determined using 30 berries per plot, which were frozen and stored at -20°C. The berry skins were removed using tweezers, taking care to remove only the skin, without pulp. The skins were washed once with water, followed by washing in deionized water and drying with absorbent paper. A 5-g skin sample was then placed in a polystyrene tube containing 50 mL of acidified methanol (1% HCl) and stored in the dark for 48 h at room temperature. The tubes were then removed from the dark and stirred manually for 5 s. Absorbance was determined using a Genesys 10S spectrophotometer (Thermo Fisher Scientific Inc., MA, United States) at 520 nm with the solvent as blank. The results were expressed in milligram malvidin-3-glucoside per gram of skin (mg/g; Peppi et al., 2006).

The CIRG was determined using 10 berries per plot with a CR-10 colorimeter (Konica Minolta, Japan), using the CIELAB color system. The following variables were determined for the berry equatorial section: lightness (*L^∗^*), saturation (*C^∗^*), and hue (*h*°). CIRG was then determined using the following equation: CIRG = (180 - *h*°)/(*L^∗^* + *C^∗^*) (Carreño et al., 1995).

Total polyphenol determination was performed using 30 berries per plot based on a modified Folin–Ciocalteu method. In summary, the absorbance of each sample was measured after 15 min at 765 nm using a Genesys 10S spectrophotometer (Thermo Fisher Scientific Inc., MA, United States) against a blank sample prepared with water instead of the extract. Determination of total polyphenol was calculated from the calibration curve obtained with gallic acid. The results were expressed in milligram total polyphenols per 100 g of sample (mg/100 g; Bucic-Kojic et al., 2007).

The berry firmness was performed with a TA.XT2i Texture Analyzer (Stable Micro Systems, Surrey, United Kingdom), at 25 ± 1°C, analyzing the equatorial position of 10 berries with pedicels per plot. Each berry was placed on the base of the equipment and compressed using a cylindrical probe with a diameter of 35 mm parallel to the base. A constant force of 0.05 N at a speed of 1.0 mm/s was applied to promote the cracking of the sample. The berry firmness (N) was then determined (Borges et al., 2012).

### Gene Expression Analyses

In the 2015 season, three grape clusters of uniform size and at the same phenological stage were identified in each plot, and two berries were collected randomly from each bunch (*n* = 6 per plot) at each sampling time. The berry skins were removed, frozen, kept at -80°C, and transferred to the Department of Viticulture and Enology of the University of California, Davis, CA, United States, for further analyses. The skins were then placed in liquid nitrogen and ground using a TissueLyser II (Qiagen, CA, United States).

RNA was extracted using 0.5 g of ground tissue (skin) based on the protocol described by Blanco-Ulate et al. (2013). RNA concentration and purity were determined using a NanoDrop 2000c Spectrophotometer (Thermo Fisher Scientific Inc., DE, United States), and RNA integrity was checked by electrophoresis on 1.5% agarose gel. Reverse transcription (synthesis of the cDNA first strand) was performed using 1 μg of RNA and M-MLV Reverse Transcriptase (Promega Corp., Madison, WI, United States) according to the manufacturer’s instructions. qRT-PCRs were performed using the SYBR^®^ Green PCR Master Mix kit (Applied Biosystems^®^, CA, United States). The PCR program consisted of 70°C for 10 min, 36 cycles at 42°C for 2 s, and 37°C for 50 min. *VvActin* (*VIT_04s0044g00580*) was used as the reference gene and processed in parallel with the genes of interest. Gene sequences used for primer design were obtained from the GenBank of the National Center for Biotechnology Information using Primer-BLAST software (Ye et al., 2012; **Table 1**). The relative levels of target gene expression were calculated using the formula 2^(Reference gene CT – Gene of interest CT)^. The linearized values correspond to the relative gene expression within a given sample and are comparable across genes. Four biological replicates of *S*-ABA treated and control grape berries were used to obtain the relative gene expression data.

**Table 1:** Primers designed for quantitative PCR analyses.

Gene name	Accession number (NCBI)	Sequence of forward (F) and reverse (R) primers		TM (melting temperature)
*CHI*	FJ468358	F	5′-CAGTCACCGCAGTTCAGGTC-3′	64.5
		R	5′-GGAAGAGGTCGTTGGTGGAG-3′	64.5
*F3H*	X75965	F	5′-ATGGCGCCTACGACACTGAC-3′	64.5
		R	5′-ATGGCTGGAAACGATGAAGCC-3′	62.6
*DFR*	pBS510	F	5′-ACCTGTAGATGGCAAGACCTAGA-3′	60.3
		R	5′-GAACTCTCATTTCCGGCACATTG-3′	60.4
*LDOX*	NM_001281218	F	5′-GACAGCTTGAGTGGGAGGAC-3′	60.4
		R	5′-AGTCGCTTGGTGTCTTAGGC-3′	58.4
*UFGT*	JF522529	F	5′-TGGTGGCTGACGCATTCAT-3′	60.2
		R	5′-CCCCATCTCTGCTGCCATATC-3′	64.5
*VvMYBA1*	AB097923	F	5′-TTATCGCAAGCCTCAGGACAG-3′	62.6
		R	5′-TCCCAGAAGCCCACATCAA-3′	60.2
*VvMYBA2*	AB073013	F	5′-GATGTGGGCTTCTGGGATAC-3′	62.4
		R	5′-AGGGAGTAGAGTATGAATGCAAGA-3′	61.2

### Anthocyanin Profiling

The same berry samples used for gene expression analyses were used for quantification of individual anthocyanins using high-pressure liquid chromatography (HPLC). A total of 0.1 g of ground tissue (skin) for each treatment and control was freeze-dried and the anthocyanins were extracted using the protocol described in Chassy et al. (2012). Four biological replicates were used to quantify the main grape anthocyanins: delphinidin-3-glucoside, cyanidin-3-glucoside, petunidin-3-glucoside, peonidin-3-glucoside, and malvidin-3-glucoside.

## Results

Application of abscisic acid (*S*-ABA) increased the total anthocyanin concentration in berry skins of the seedless grape Selection 21 during the 2013 and 2014 seasons, regardless of the *S*-ABA concentration and time of application (**Table 2**). However, berries that received 400 mg/L of *S*-ABA at 7 and 21 DAV had significantly higher, almost two to three times more, anthocyanin concentrations than other treatments.

**Table 2:** Total anthocyanin, color index for red grapes (CIRG), and color coverage of Selection 21 grape berries treated with *S*-ABA.

Treatment (concentration in mg/L)	Total anthocyanin (mg/g)		CIRG		Color coverage^1^	
	2013	2014	2013	2014	2013	2014
Control	0.18 ± 0.07 d	0.12 ± 0.03 d	1.49 ± 0.49 c	1.80 ± 0.12 b	1.00 ± 0.00 d	1.00 ± 0.00 c
*S-*ABA 200 (7 DAV)	0.39 ± 0.14 cd	0.59 ± 0.14 c	2.15 ± 0.29 bc	3.71 ± 0.61 a	1.75 ± 0.50 c	3.00 ± 0.50 b
*S-*ABA 400 (7 DAV)	0.57 ± 0.15 bc	0.86 ± 0.06 b	2.78 ± 0.53 b	4.48 ± 0.28 a	3.25 ± 0.50 b	4.25 ± 0.75 ab
*S-*ABA 200 (7 DAV) + 200 (21 DAV)	0.68 ± 0.02 b	0.74 ± 0.04 bc	3.07 ± 0.33 b	4.34 ± 0.13 a	2.75 ± 0.50 b	3.75 ± 0.38 ab
*S-*ABA 400 (7 DAV) + 400 (21 DAV)	1.15 ± 0.04 a	1.18 ± 0.01 a	4.14 ± 0.54 a	4.51 ± 0.13 a	4.75 ± 0.50 a	5.00 ± 0.00 a

*Mean values within columns followed by different letters differ significantly at the 5% probability level according to Tukey’s test. DAV, days after véraison. ^1^Color coverage: (1) 0–20%; (2) 21–40%; (3) 41–60%; (4) 61–80%; (5) 81–100% coverage. 2013 and 2014 seasons.*

**FIGURE 2:**
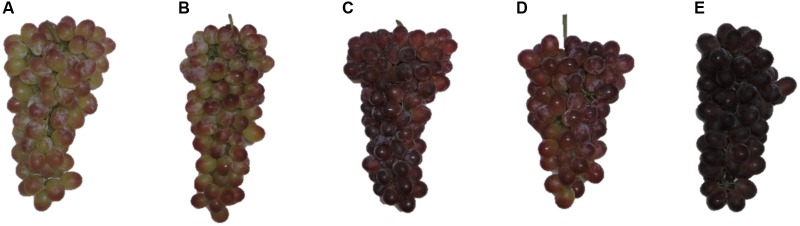
Representative clusters of seedless grape Selection 21 subjected to various treatments with *S*-ABA. **(A)** Control; **(B)** *S*-ABA 200 mg/L 7 days after véraison (DAV); **(C)** *S*-ABA 400 mg/L 7 DAV; **(D)** *S*-ABA 200 mg/L 7 DAV + 200 mg/L 21 DAV; **(E)** *S*-ABA 400 mg/L 7 DAV + 400 mg/L 21 DAV.

According to the CIRG, berries from control treatments had a green to a yellow color (CIRG < 2) in both seasons (**Table 2**). In 2013, berries treated with one or two applications of 200 mg/L *S*-ABA or one application of 400 mg/L *S*-ABA at 7 DAV, and those in the 2014 season that were treated with one application of 200 mg/L *S*-ABA developed a pink color (2 < CIRG < 4). Remarkably, berries of the 2013 season treated with two applications of 400 mg/L *S*-ABA and berries of the 2014 season treated with one or two 400 mg/L *S*-ABA applications, developed a stronger red color (4 < CIRG < 5; **Figure 2**). For both the 2013 and 2014 seasons, color coverage was lowest in control grapes and highest in grapes treated with two applications of 400 mg/L *S*-ABA.

Increase in total polyphenols was evident in grapes subjected to two 400 mg/L *S*-ABA applications during the 2013 and 2014 seasons. These berries also presented the lowest mean berry firmness (**Table 3**). Importantly, the increased softening due to *S*-ABA application did not result in higher frequency of cracked berries in any of the studied seasons. Qualitative assessment of berry cracking was performed visually.

**Table 3:** Total polyphenols and firmness of Selection 21 grape berries treated with *S*-ABA.

Treatment (concentration in mg/L)	Total polyphenol content (mg/100 g)		Firmness (N)	
	2013	2014	2013	2014
Control	15.9 ± 3.8	13.4 ± 2.5 b	33.9 ± 5.0 a	25.3 ± 2.5 a
*S-*ABA 200 (7 DAV)	13.1 ± 3.0	12.8 ± 1.5 b	30.2 ± 3.1 a	23.7 ± 1.3 a
*S-*ABA 400 (7 DAV)	15.5 ± 1.7	14.3 ± 0.7 b	26.7 ± 2.9 b	19.3 ± 1.9 bc
*S-*ABA 200 (7 DAV) + 200 (21 DAV)	15.8 ± 1.9	14.4 ± 1.2 b	26.4 ± 1.3 b	20.4 ± 1.6 bc
*S-*ABA 400 (7 DAV) + 400 (21 DAV)	18.3 ± 1.1	18.3 ± 1.3 a	19.9 ± 2.0 c	15.7 ± 0.5 c

*Mean values within columns followed by different letters differ significantly at the 5% probability level according to Tukey’s test. DAV, days after véraison. 2013 and 2014 seasons.*

**FIGURE 3:**
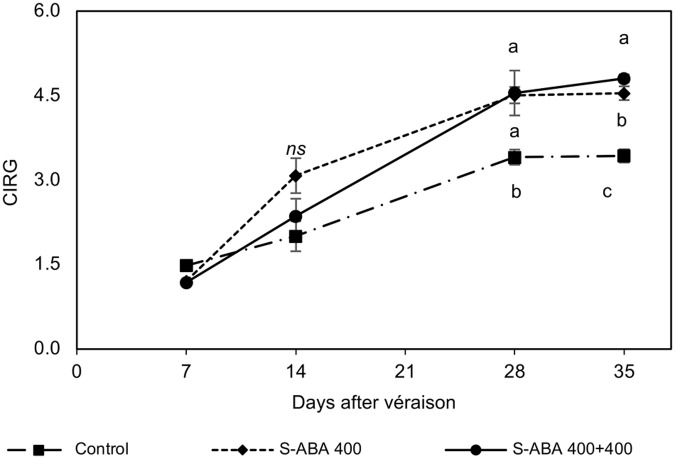
CIRG of Selection 21 grape berries subjected to treatments with *S*-ABA during the 2015 season. Control: no *S*-ABA application; *S*-ABA 400: *S*-ABA 400 mg/L 7 days after véraison (DAV); *S*-ABA 400 + 400: *S*-ABA 400 mg/L 7 DAV + 400 mg/L 21 DAV. The means followed by different letters differ significantly at the 5% probability level according to Tukey’s test. ns, non-significant.

Further analyses of the effect of 400 mg/L *S*-ABA treatments on CIRG, total and individual anthocyanins concentrations, and gene expression of transcription factors and biosynthetic enzymes were performed with grape berries collected from the 2015 trial. Measurements of CIRG confirmed previous results obtained during the 2013 and 2014 seasons, at the time of harvest (35 DAV), grapes treated with two *S*-ABA applications had the highest CIRG values (**Figure 3**). Grape bunches from the control treatment presented pink berries (CIRG = 3.4), whereas those treated with one or two applications of *S*-ABA had red berries (CIRG = 4.5 and CIRG = 4.8, respectively). As determined in the previous seasons, berries treated with 400 mg/L *S*-ABA also presented higher total anthocyanin content than the control at 14 and 28 DAV (**Table 4**). At 28 DAV, grapes treated with one (7 DAV) or two (7 and 21 DAV) applications of 400 mg/L *S*-ABA presented total anthocyanin concentrations almost three times higher than the control. Even 3 weeks after the first application (28 DAV), berries treated with only one *S*-ABA application showed a total anthocyanin content similar to those treated with two *S*-ABA applications. Nonetheless, the second application of 400 mg/L *S*-ABA significantly affected the total anthocyanin accumulation at the time of harvest (35 DAV, 4 weeks after the first *S*-ABA application and 2 weeks after the second *S*-ABA application).

**Table 4:** Total and individual anthocyanin content of Selection 21 grape berries treated with *S*-ABA during 2015 season.

Sampling	Treatment (concentration in mg/L)	Total anthocyanin content (mg/g)	Anthocyanin content (mg/L)				
			Delphinidin-3-glucoside	Cyanidin-3-glucoside	Petunidin-3-glucoside	Peonidin-3-glucoside	Malvidin-3-glucoside
7 DAV	Control	0.07 ± 0.01	-	-	2.53 ± 0.43	-	-
	*S*-ABA 400 (7 DAV)	0.06 ± 0.01	-	-	2.05 ± 0.38	-	-
	*S*-ABA 400 (7 DAV) + 400 (21 DAV)	0.06 ± 0.01	-	-	1.53 ± 0.39	-	-
14 DAV	Control	0.23 ± 0.09b	-	0.08 ± 0.32c	3.07 ± 0.83	1.33 ± 0.48b	-
	*S*-ABA 400 (7 DAV)	0.50 ± 0.13a	-	4.85 ± 0.43a	2.35 ± 1.00	7.80 ± 0.05a	-
	*S*-ABA 400 (7 DAV) + 400 (21 DAV)	0.40 ± 0.13a	-	2.80 ± 0.86b	2.08 ± 0.07	5.10 ± 1.60a	-
28 DAV	Control	0.56 ± 0.17b	0.10 ± 0.01b	7.53 ± 2.33b	2.03 ± 0.33	12.57 ± 4.94b	0.23 ± 0.88b
	*S*-ABA 400 (7 DAV)	1.54 ± 0.08a	0.12 ± 0.03b	20.58 ± 1.01a	1.40 ± 0.58	45.93 ± 3.48a	3.13 ± 0.98a
	*S*-ABA 400 (7 DAV) + 400 (21 DAV)	1.57 ± 0.05a	0.38 ± 0.03a	26.23 ± 2.43a	0.95 ± 0.57	40.03 ± 7.17a	2.40 ± 0.01a
35 DAV	Control	0.70 ± 0.04c	0.10 ± 0.01c	9.70 ± 0.77c	1.60 ± 0.94	15.88 ± 2.91c	0.25 ± 0.06b
	*S*-ABA 400 (7 DAV)	1.52 ± 0.02b	1.52 ± 0.50b	21.58 ± 1.82b	1.00 ± 0.13	46.40 ± 1.86b	5.35 ± 1.58a
	*S*-ABA 400 (7 DAV) + 400 (21 DAV)	1.65 ± 0.01a	3.11 ± 0.62a	29.70 ± 1.19a	0.93 ± 0.23	54.47 ± 2.36a	4.60 ± 2.07a

*Mean values within columns followed by different letters differ significantly at the 5% probability level according to Tukey’s test. ns, non-significant. Control, no S-ABA application; 400, S-ABA 400 mg/L 7 days after véraison (DAV); 400 + 400, S-ABA 400 mg/L at 7 DAV + 400 mg/L S-ABA at 21 DAV. –, not detected.*

*S*-ABA altered the concentrations and proportions of individual anthocyanins in berries from the seedless grape Selection 21 (**Table 4**). With the exception of petunidin-3-glucoside, *S*-ABA application significantly improved the concentrations of all the measured anthocyanins. Cyanidin-3-glucoside and peonidin-3-glucoside levels increased at 14 DAV, 1 week after the first *S*-ABA application. The second *S*-ABA application stimulated the accumulation of the anthocyanin delphinidin-3-glucoside at 28 DAV, yielding differences relative to both the control and to the samples treated with only one *S*-ABA application. At 28 DAV, the concentrations of peonidin-3-glucoside and malvidin-3-glucoside increased after exogenous *S*-ABA application but were not further increased by the second application. At the time of harvest (35 DAV), peonidin-3-glucoside and cyanidin-3-glucoside were the dominant pigments present after all treatments. Delphinidin-3-glucoside, cyanidin-3-glucoside, and peonidin-3-glucoside presented higher accumulation following the second application of 400 mg/L *S*-ABA, but the number of applications did not affect the accumulation of malvidin-3-glucoside.

**FIGURE 4:**
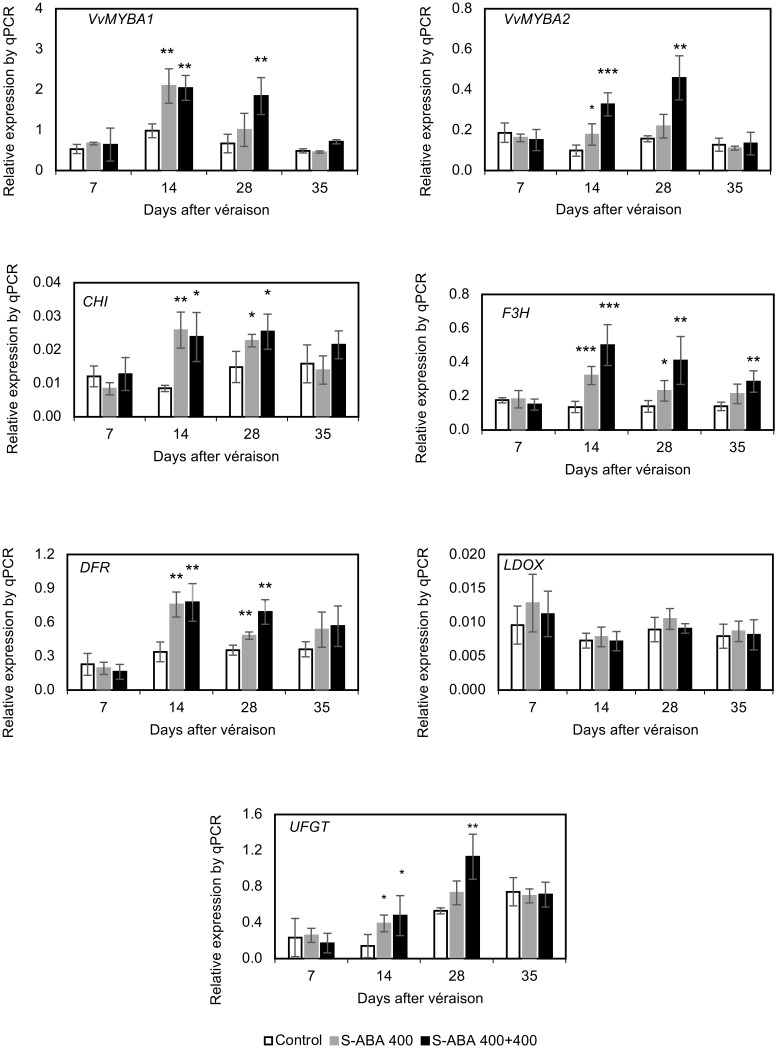
Effects of *S*-ABA application on the expression of *VvMYBA1* and *VvMYBA2* and the transcription levels of the flavonoid biosynthetic genes in grape berries from the Selection 21 hybrid throughout development in the 2015 season. Student’s *t*-test was used to determine significant differences between the treatments and the control at ^∗^*p* ≤ 0.05, ^∗∗^*p* ≤ 0.01, and ^∗∗∗^*p* ≤ 0.001. Control, no *S*-ABA application; *S*-ABA 400, *S*-ABA 400 mg/L 7 days after véraison (DAV); *S*-ABA 400 + 400, *S*-ABA 400 mg/L at 7 DAV + 400 mg/L at 21 DAV.

As presented in **Figure 4**, treatment with 400 mg/L *S*-ABA significantly increased the expression of the transcription factors *VvMYBA1* and *VvMYBA2* and the expression of the biosynthetic genes *CHI*, *F3H*, *DFR*, and *UFGT* 1 week after the first application (14 DAV). Three weeks after the first *S*-ABA application (28 DAV), expression of *CHI*, *F3H*, and *DFR* genes remained high, but this was not observed for the transcription factors *VvMYBA1* and *VvMYBA2* or the *UFGT* gene. Four weeks after the first *S*-ABA application (35 DAV), no significant differences were observed in the expression of genes or transcription factors between berries that received one *S*-ABA application and those that received the control treatment. The two applications of 400 mg/L *S*-ABA induced expression of the genes *CHI*, *F3H*, *DFR*, and *UFGT* and the transcription factors *VvMYBA1* and *VvMYBA2* at 14 and 28 DAV (**Figure 4**). *F3H* expression was the most affected by *S*-ABA application, displaying higher levels than the control until the final stages of berry maturation at 35 DAV, whereas the remaining genes presented no differences from the control at harvest. Overall, the gene expression results indicate that a second *S*-ABA application contributed to the maintenance of the expression of the transcription factors *VvMYBA1* and *VvMYBA2* and the genes *F3H* and *UFGT* at higher levels than in the control for an extended period of time.

## Discussion

### Effects of *S*-ABA on Anthocyanin Concentration, Berry Color, and Firmness

Exogenous application of *S*-ABA improves the color of table grapes by stimulating the anthocyanin synthesis and accumulation in the grape skin (Jeong et al., 2004; Cantín et al., 2007). Our results are similar to those reported previously for “Flame Seedless” grapes, in which applications of 300 mg/L ABA during or after véraison were more effective at increasing anthocyanin concentrations than application before véraison (Peppi et al., 2006). In ‘Crimson Seedless’ grapes, anthocyanin concentrations increased with application of 400 mg/L *S*-ABA at 17 DAV, but the response varied widely between seasons depending on *S*-ABA dosage and time of application (Leão et al., 2015). Exogenous *S*-ABA application is thought to simulate plant stress responses and accelerate ripening processes (Balint and Reynolds, 2013). High ABA concentrations are believed to be perceived by grapevines as a drought stress signal (Castellarin et al., 2007). Subsequently, water stress leads to changes in grape secondary metabolism, significantly increasing flavonoid levels and, especially, anthocyanin biosynthesis (Zhang et al., 2016).

In our study, in addition to increasing anthocyanin concentration, exogenous *S*-ABA improved both berry color intensity and uniformity. This is important because the visual assessment of berry color characteristics determine, in part, the commercial value of table grapes (Peppi et al., 2006, 2007). Grape clusters with more intense and uniform berry color have higher consumer acceptance. Improved color characteristics such as increased color coverage of grape berries, and uniformity of berry color within a cluster, were also observed in “Benitaka” and “Rubi” table grapes following application of 400 mg/L *S*-ABA at 7 DAV and 15 days before harvest (Roberto et al., 2012, 2013). In our experiments, berries from the seedless grape Selection 21 treated with one or two applications of 400 mg/L *S*-ABA presented higher CIRG values than the controls. Besides, improving cluster color and attractiveness, exogenous *S*-ABA can potentially decrease the time to harvest, a feature that is very advantageous for grape commercialization (Cantín et al., 2007; Ferrara et al., 2015).

The observed increase in anthocyanin concentration resulting from *S*-ABA application does not necessarily result in an increase in total polyphenol content; polyphenols include phenolic acids, stilbenes, coumarins, tannins, and flavonoids (Roubelakis-Angelakis, 2009), as reported in “Alachua” muscadine grapes (Sandhu et al., 2011) and in “Isabel” grapes (Koyama et al., 2014). Environmental factors such as temperature, rainfall, and altitude could also influence berry polyphenol concentrations. In addition, the berry ripening stage is directly correlated to the concentration and proportion of several polyphenols that impact the organoleptic properties, nutritional value, and antioxidant capacities of the grapes (Zhu et al., 2016).

*S*-ABA application can negatively affect berry firmness, an important characteristic for the successful postharvest handling of grapes for the fresh fruit market because it influences transportability and shelf life (Batista et al., 2015). ABA application is known to cause loosening and decreased rigidity of the cell wall, resulting in fruit softening and a higher probability of berry cracking (Thomas et al., 2008; Wada et al., 2008; Gambetta et al., 2010). Treatment of grapes with exogenous *S*-ABA can result in changes in the regulation of proline-rich cell wall proteins and in the induction of cell wall degrading genes, such as polygalacturonases that promote pectin solubilization and depolymerization (Koyama et al., 2010). The effect of *S*-ABA on berry firmness was also observed in “Flame Seedless” grapes, where it caused softening similar to that caused by ethephon application (Peppi et al., 2006), as well as in ‘Crimson Seedless’ (Lurie et al., 2009) and “Red Globe” grapes (Peppi et al., 2007). Therefore, it is still necessary to evaluate if the benefits of applying exogenous ABA to improve berry color can outweigh a potential reduction in the shelf life of treated grapes.

### The Effect of Multiple Applications of *S*-ABA on Individual Anthocyanins Concentration, Gene Expression, and Transcript Factors Expression

Multiple applications of exogenous ABA can promote anthocyanin accumulation for longer periods of time (Giribaldi et al., 2010). It is possible that more than one ABA application could induce a milder response at later grape phenological stages or that the effects of a second application could take more time to be evident. In this study, the second application of 400 mg/L *S*-ABA significantly increased the total anthocyanin content at the time of harvest, which supported the latter hypothesis and confirmed that two *S*-ABA applications had a more pronounced effect than only one application. The higher total anthocyanin concentration observed with *S*-ABA application appeared to result from a transient effect of *S*-ABA, because the anthocyanin concentration of grapes that received only one *S*-ABA application remained essentially constant between 28 and 35 DAV. It may, therefore, be inferred that the action of *S*-ABA decreases over time and that its levels increase with a second application, allowing maintenance of its activity.

Three applications of 400 mg/L *S*-ABA at 1-week intervals prior to véraison resulted in an earlier accumulation of anthocyanin in “Cabernet Sauvignon” grapes, but no differences in anthocyanin concentration at harvest were observed in grapes that received different treatments (Wheeler et al., 2009). The increase in endogenous ABA concentration in grape berries occurs at the beginning of véraison and extends until the establishment of maturation when endogenous ABA concentrations peak. ABA concentration then decreases until harvest, the period over which the decrease occurs ranges from 13 to 20 days depending on the cultivar (Davies et al., 1997; Wheeler et al., 2009). Application of exogenous *S*-ABA close to véraison, when ABA naturally reaches its highest concentration in berries, was shown to be more effective in increasing anthocyanin accumulation than application at other times (Berli et al., 2011). *S*-ABA application significantly increased endogenous ABA levels 7 days after application in “Carménère” grapes, 40 days later, the ABA levels in the treated berries remained higher than those from control (Villalobos-González et al., 2016).

Berry color is affected both by total anthocyanin concentration and by anthocyanin composition. Different anthocyanins have specific characteristics with respect to color and stability (Jeong et al., 2004). The five main grape anthocyanins differ from each other in the number and position of the hydroxyl (OH) and methyl (CH_3_) groups on the B-ring. Cyanidin and peonidin are dihydroxylated precursors of red anthocyanins in grape skin, whereas delphinidin, petunidin, and malvidin are trihydroxylated precursors of blue and purple anthocyanins (Liang et al., 2009; Azuma et al., 2015). Accumulation of individual anthocyanins in grapes may be induced by *S*-ABA application and varies with the cultivar. In “Noble” and “Alachua” muscadine grapes, application of *S*-ABA during véraison and again at 8 DAV for “Noble” or again at 13 DAV for “Alachua” resulted in higher levels of accumulation of all evaluated anthocyanins in “Noble” grapes but not in “Alachua” grapes, which only presented higher accumulation of peonidin-3-diglucoside compared to the control (Sandhu et al., 2011). Changes in the proportions of individual anthocyanins resulting from *S*-ABA application were also observed in “Cabernet Sauvignon” grapes, both in berries and in wine (González et al., 2012). In “Isabel” grapes, application of *S*-ABA increased the accumulation of individual anthocyanins both in must and in processed whole juice (Yamamoto et al., 2015). Similar results were observed in wines prepared from “Merlot” grapes treated with a racemic mixture of ABA (*S*-ABA and *R*-ABA). This treatment resulted in changes in the proportions of anthocyanins, increased total phenol and flavonol content, and increased antioxidant activity (Koyama et al., 2014). However, it should be considered that application of racemic mixtures of enantiomers may result in a range of plant responses because *R*-ABA is not found in plants and is less active and less effective than *S*-ABA. The two enantiomeric forms may have different effects on gene expression and on physiological responses (Lacampagne et al., 2010; Zhang, 2014).

Anthocyanin accumulation in grape berries during véraison is probably triggered by increased sugar and ABA concentrations in the berry skin, which activate the expression of genes involved in anthocyanin biosynthesis (Castellarin et al., 2015). The activation threshold for genes involved in anthocyanin production was reported to be between 9 and 10°Bx (Keller, 2015). *S*-ABA application at 7 DAV, when anthocyanin biosynthetic genes are normally induced, followed by a second application at 21 DAV, when endogenous ABA concentrations are close to maximal or are beginning to decrease, can upregulate their expression even further or maintain them at a constant level for a longer period of time.

Anthocyanins are produced through multiple pathways that are controlled by MYB transcription factors. These transcription factors are responsive to ABA and are associated with the regulation of the biosynthetic genes *CHI*, *F3H*, *DFR*, *LDOX*, and *UFGT* (Yu et al., 2012; Oglesby et al., 2016; Olivares et al., 2017). The transcription factors *VvMYBA1* and *VvMYBA2* activate anthocyanin biosynthesis in grapevines and are not functional in white grape cultivars (Rinaldo et al., 2015). Transcription factors affect the ratio of tri-/dihydroxylated anthocyanins through trans-regulation of flavonoid 3-hydroxylase (*F3′H*) and flavonoid 3′5′-hydroxylase (*F3′,5′H*) gene expression (Owen et al., 2009). During anthocyanin biosynthesis, F3H is responsible for the hydroxylation of naringenin at position 3′, generating dihydrokaempferol, a dihydroflavonol that can be hydrolyzed at position 3′ or 5′ of the B-ring by the enzymes F3′H or F3′,5′H, which are responsible for the hydroxylation of the B-ring of flavonoids. F3′H activity promotes accumulation of the cyanidin and peonidin anthocyanin groups, whereas F3′,5′H activity results in the production of delphinidin and its derivatives petunidin and malvidin. These two enzymes compete in controlling di- and trihydroxylated anthocyanin synthesis (Roubelakis-Angelakis, 2009). In our study, treatment of hybrid grapes with two applications of 400 mg/L *S*-ABA primarily favored the accumulation of delphinidin-3-glucoside and malvidin-3-glucoside (**Table 4**); therefore, such treatment decreased the difference between the concentrations of di- and trihydroxylated anthocyanins in the grapes. This is consistent with previous results obtained for “Aki Queen” grapes (*Vitis labruscana*), in which the application of *S*-ABA stimulated the gene expression of *F3′,5′H* relative to *F3′H*. In addition, the concentrations of petunidin and malvidin increased in the berries, thereby increasing the proportion of trihydroxylated anthocyanins and decreasing the proportions of cyanidin and peonidin anthocyanins relative to the total anthocyanins (Katayama-Ikegami et al., 2016). In this study, the expression of the main enzymes leading to anthocyanin biosynthesis were analyzed. Future experiments to study changes in expression of F3′H and F3′,5′H encoding genes are still required to gain a better insight into the impact of exogenous ABA applications on the differential accumulation of specific anthocyanins.

Our results indicate that application of *S*-ABA increased the expression of the *UFGT* gene and the transcription factors at 28 DAV, but this was not observed for the treatment with only one *S*-ABA application. Anthocyanin accumulation begins when all genes involved in the biosynthetic pathway are induced, especially *UFGT* (Davies et al., 1997). Anthocyanidins (anthocyanins lacking sugar moieties) are unstable and are easily degraded to colorless compounds; therefore, before anthocyanins are transported, they must be stabilized by the addition of a glucose residue at position 3 of the C-ring (Castellarin et al., 2007). The enzyme UFGT catalyzes the final step of anthocyanin biosynthesis, therefore UFGT has been considered by many authors to be a critical enzyme in anthocyanin biosynthesis (Davies et al., 1997).

Temporary stimulation of gene transcription is believed to be related to a decrease in *S*-ABA concentration over time. In ‘Crimson Seedless’ grapes, a constant decrease in *S*-ABA levels with a half-life time of 14.7 days was observed in treated grape berries (Ferrara et al., 2013). The natural decrease in ABA concentration, along with the decrease in *S*-ABA levels, may, therefore, lead to decreased activity of some genes, depending on the *S*-ABA concentration in the plant. Expression of the *UFGT* gene increased considerably 7 days after *S*-ABA application in ‘Crimson Seedless’ grapes but decreased 3 weeks after treatment, becoming similar to the control (Koyama et al., 2010). In “Cabernet Sauvignon” grapes treated with ±*cis*, *trans*-ABA, expression analysis of anthocyanin biosynthetic genes revealed that the maximum expression levels were only reached 10–17 days after application and that they then rapidly decreased (Lacampagne et al., 2010). ABA *cis*- and *trans*-isomers differ in the orientation of the carboxyl group at carbon 2. Only the ABA *cis*-isomer is biologically active, and it accounts for almost all of the ABA produced in plant tissues. However, unlike the *S* and *R* enantiomers, the *cis*- and *trans*-isomers can be interconverted in plant tissue (Keller, 2015).

Most of the studies on *S*-ABA involved *V. vinifera* cultivars were done in temperate zones and testing a single application (Peppi et al., 2006, 2008; Koyama et al., 2010; Ferrara et al., 2015; Zhu et al., 2016). In this study, we evaluated the response of a new *V. vinifera* ×*V. labrusca* hybrid grape cultivar grown in a subtropical area to multiple *S*-ABA applications. This hybrid often shows lack of color development; therefore, our results confirm the effectiveness of *S*-ABA to improve the color of ripening berries, even under warm climate conditions. The application of *S*-ABA to berries of the seedless grape Selection 21 increased the total anthocyanin concentration, changed the proportion of individual anthocyanins, improved their color attributes, and increased the expression of transcription factors and anthocyanin biosynthetic genes. Two applications of 400 mg/L *S*-ABA, at 7 and 21 DAV, resulted in the best results in terms of color increment and total anthocyanin concentration, favored the accumulation of trihydroxylated anthocyanins, and increased the expression of transcription factors and of the genes *F3H* and *UFGT*.

These results not only show that *S*-ABA is a valuable tool for improving the color of red grapes in warm areas, where color deficiency is frequently observed, but also suggest that *S*-ABA may be useful in grape breeding programs by permitting the selection and release of new cultivars with natural poor color, but other desirable characteristics such as high yield and resistance to common diseases.

## Conclusion

Exogenous application of *S*-ABA increased the total anthocyanin accumulation in the berry skin of hybrid grapes. Two applications of 400 mg/L *S*-ABA, at 7 and 21 DAV, resulted in increased concentrations of the anthocyanins delphinidin-3-glucoside, cyanidin-3-glucoside, peonidin-3-glucoside, and malvidin-3-glucoside, increased expression of the biosynthetic genes *CHI*, *F3H*, *DFR*, and *UFGT*, and increased expression of the transcription factors *VvMYBA1* and *VvMYBA2*.

## Author Contributions

RK, SR, RdS, AW, DC, MF, and BB-U conceived and designed the experiments. RK, SR, and WB performed the experiments. RK, SR, DC, MF, and BB-U wrote the manuscript. RK, MA, WB, and BB-U analyzed the data. All authors have read and approved the final version of the manuscript.

## Conflict of Interest Statement

The authors declare that the research was conducted in the absence of any commercial or financial relationships that could be construed as a potential conflict of interest.
